# IgM antibodies against malondialdehyde and phosphorylcholine in different systemic rheumatic diseases

**DOI:** 10.1038/s41598-020-66981-z

**Published:** 2020-07-03

**Authors:** Divya Thiagarajan, Nina Oparina, Susanna Lundström, Roman Zubarev, Jitong Sun, The PRECISESADS Clinical Consortium, Lorenzo Beretta, Barbara Vigone, Jacques-Olivier Pers, Alain Saraux, Valérie Devauchelle-Pensec, Divi Cornec, Sandrine Jousse-Joulin, Bernard Lauwerys, Julie Ducreux, Anne-Lise Maudoux, Carlos Vasconcelos, Ana Tavares, Esmeralda Neves, Raquel Faria, Mariana Brandão, Ana Campar, António Marinho, Fátima Farinha, Isabel Almeida, Miguel Angel Gonzalez-Gay Mantecón, Ricardo Blanco Alonso, Alfonso Corrales Martínez, Ricard Cervera, Ignasi Rodríguez-Pintó, Gerard Espinosa, Rik Lories, Ellen De Langhe, Nicolas Hunzelmann, Doreen Belz, Torsten Witte, Niklas Baerlecken, Georg Stummvoll, Michael Zauner, Michaela Lehner, Eduardo Collantes, Rafaela Ortega-Castro, Mª Angeles Aguirre-Zamorano, Alejandro Escudero-Contreras, Mª Carmen Castro-Villegas, Norberto Ortego, María Concepción Fernández Roldán, Enrique Raya, Inmaculada Jiménez Moleón, Enrique de Ramon, Isabel Díaz Quintero, Pier Luigi Meroni, Maria Gerosa, Tommaso Schioppo, Carolina Artusi, Carlo Chizzolini, Aleksandra Zuber, Donatienne Wynar, Laszló Kovács, Attila Balog, Magdolna Deák, Márta Bocskai, Sonja Dulic, Gabriella Kádár, Falk Hiepe, Velia Gerl, Silvia Thiel, Manuel Rodriguez Maresca, Antonio López-Berrio, Rocío Aguilar-Quesada, Héctor Navarro-Linares, Marta Alarcon-Riquelme, Johan Frostegård

**Affiliations:** 1 Unit of Immunology and Chronic Disease, Institute of Environmental Medicine, Karolinska Institutet, Stockholm, Sweden; 2 Division of Physiological Chemistry I, Department of Medical Biochemistry and Biophysics, Karolinska Institutet, Stockholm, Sweden; 3 GENYO, Center for Genomics and Oncological Research: Pfizer/University of Granada/Andalusian Government, Parque tecnolуgico de la salud, 18016 Granada, Spain; 4 Referral Center for Systemic Autoimmune Diseases, Fondazione IRCCS Ca’ Granda Ospedale Maggiore Policlinico di Milano, Milan, Italy; 5 Centre Hospitalier Universitaire de Brest, Hospital de la Cavale Blanche, Brest, France; 6 Pôle de pathologies rhumatismales systémiques et inflammatoires, Institut de Recherche Expérimentale et Clinique, Université catholique de Louvain, Brussels, Belgium; 7 Centro Hospitalar do Porto, Porto, Portugal; 8 Servicio Cantabro de Salud, Hospital Universitario Marqués de Valdecilla, Santander, Spain; 9 Hospital Clinic I Provicia, Institut d’Investigacions Biomèdiques August Pi i Sunyer, Barcelona, Spain; 10 Katholieke Universiteit Leuven, Leuven, Belgium; 11 Klinikum der Universitaet zu Koeln, Cologne, Germany; 12 Medizinische Hochschule Hannover, Hanover, Germany; 13 Medical University Vienna, Vienna, Austria; 14 Servicio Andaluz de Salud, Hospital Universitario Reina Sofía Córdoba, Córdoba, Spain; 15 Servicio Andaluz de Salud, Complejo hospitalario Universitario de Granada (Hospital Universitario San Cecilio), Granada, Spain; 16 Servicio Andaluz de Salud, Complejo hospitalario Universitario de Granada (Hospital Virgen de las Nieves), Granada, Spain; 17 Servicio Andaluz de Salud, Hospital Regional Universitario de Málaga, Málaga, Spain; 18 Università degli studi di Milano, Milan, Italy; 19 Hospitaux Universitaires de Genève, Geneva, Switzerland; 20 University of Szeged, Szeged, Hungary; 21 Charite, Berlin, Germany; 22 Andalusian Public Health System Biobank, Granada, Spain

**Keywords:** Rheumatology, Rheumatic diseases

## Abstract

IgM antibodies against phosphorylcholine (anti-PC) and malondialdehyde (anti-MDA) may have protective properties in cardiovascular and rheumatic diseases. We here compare these antibodies in systemic rheumatic conditions and study their properties. Anti-PC and anti-MDA was measured using ELISA in patients with SLE (374), RA (354), Mixed connective tissue disease (MCTD, 77), Systemic sclerosis (SSc, 331), Sjögren’s syndrome (SjS, 324), primary antiphospholipid syndrome (PAPs, 65), undifferentiated connective tissue disease (UCTD, 118) and 515 matched healthy controls (HC). Cardiovascular score (CV) was broadly defined based on clinical disease symptoms. Anti-PC and anti-MDA peptide/protein characterization were compared using a proteomics de novo sequencing approach. anti-MDA and anti-PC were extracted from total IgM. The proportion of Treg cells was determined by flow cytometry. The maximal difference between cases and controls was shown for MCTD: significantly lower IgM Anti-PC but not anti-MDA among patients (median 49.3RU/ml vs 70.4 in healthy controls, p(t-test) = 0.0037). IgM low levels were more prevalent in MCTD, SLE, SjS, SSc and UCTD. IgM anti-PC variable region profiles were different from and more homologous than anti-MDA. Anti-PC but not anti-MDA were significantly negatively correlated with CV in the whole patient group. In contrast to IgM anti-PC, anti-MDA did not promote polarization of Tregs. Taken together, Anti-PC is decreased in MCTD and also in SLE, SjS and SSc but not in other studied diseases. Anti-PC may thus differentiate between these. In contrast, anti-MDA did not show these differences between diseases studied. Anti-PC level is negatively correlated with CV in the patient group cohort. In contrast to anti-PC, anti-MDA did not promote Treg polarization. These findings could have both diagnostic and therapeutic implications, one possibility being active or passive immunization with PC in some rheumatic conditions.

## Introduction

Systemic autoimmune diseases (SADs) is present among up to 3–5% of population in westernized societies. Their pathogenesis involves varying degree of chronic inflammation but usually the underlying causes remain elusive. A common feature is autoimmune reactions and specific autoantibodies and current diagnosis is typically based on a combination of autoantibody patterns and clinically related criteria^1–3^.

In the present study, PRECISESADS (Precision medicine strategies for Systemic Autoimmune Diseases) patients with various autoimmune diseases: SLE (Systemic Lupus Erythematosus), RA (Rheumatoid Arthritis), SjS (Sjögren´s syndrome), SSc (Systemic Sclerosis), MCTD (Mixed Connective Tissue Disease), PAPs (Primary Antiphospholipid syndrome) and UCTD (Undifferentiated Connective Tissue Disease), were recruited.

The SADs patients present multiple co-morbidities, and an important one is cardiovascular diseases, often based on increased atherosclerosis^4–7^. Phosphorylcholine (PC) is a damage associated molecular pattern (DAMP)^8^ and also a pathogen associated molecular pattern (PAMP)^9,10^ while malondialdehyde (MDA) is mainly considered a DAMP^11^. Both form adducts with proteins and are recognized by the immune system. As a consequence, anti-PC and anti-MDA IgM antibodies are present at relatively high concentrations in healthy adults. Since recognized antigens are normally present in humans these IgMs could be considered autoantibodies^12–15^.

We have reported protective associations for IgM anti-PC in different chronic inflammatory disease conditions. It is negatively associated with cardiovascular disease including stroke and myocardial infarction (MI), atherosclerosis increase after four years, and also mortality after MI^12–14^. IgM anti-PC could also play a role in SLE being associated with the disease itself, and also with atherosclerotic plaques and vulnerable plaques in SLE^16,17^. Further, low anti-PC is associated with being a non-responder to biologics in RA^18^. These and similar findings have largely been confirmed and extended into other diseases like vasculitis and even osteoarthritis^19–25^.

Less is known about clinical role of anti-MDA IgM. We reported that in SLE, anti-MDA is a protection marker, especially together with anti-PC^26^ and anti-MDA is also a protection marker for development of CVD among 60-year olds^15^.

We here determine anti-PC and anti-MDA in the patients studied and compared with healthy controls. We also compare anti-PC and anti-MDA with peptide/protein characterization using a proteomics de novo sequencing approach and study effects of these antibodies on T regulatory cells (Tregs).

## Materials and Methods

In the PRECISESADS study, patients with SLE (n = 374), RA (n = 354), MCTD (n = 77), SSc (n = 331), SjS (n = 324), PAPs (n = 65), UCTD (n = 118) and 515 age- and sex-matched healthy controls (HC) were investigated. This study and classification of patients has been described previously^27^. The participants were recruited, and samples were obtained in accordance with the Declaration of Helsinki. All participants gave informed written consent to participating in the study. Ethical approval was granted at each participating site by the ethics committee/institutional review board at the following institutions:

Referral Center for Systemic Autoimmune Diseases, Fondazione IRCCS Ca’ Granda Ospedale Maggiore Policlinico di Milano, Italy;

Centre Hospitalier Universitaire de Brest, Hospital de la Cavale Blanche, Brest, France**;**

Pôle de pathologies rhumatismales systémiques et inflammatoires, Institut de Recherche Expérimentale et Clinique, Université catholique de Louvain, Brussels, Belgium**;**

Centro Hospitalar do Porto, Portugal**;**

Servicio Cantabro de Salud, Hospital Universitario Marqués de Valdecilla, Santander, Spain**;**

Hospital Clinic I Provicia, Institut d’Investigacions Biomèdiques August Pi i Sunyer, Barcelona, Spain**;**

Katholieke Universiteit Leuven, Belgium**;**

Klinikum der Universitaet zu Koeln, Cologne, Germany**;**

Medizinische Hochschule Hannover, Germany**;**

Medical University Vienna, Vienna, Austria**;**

Servicio Andaluz de Salud, Hospital Universitario Reina Sofía Córdoba, Spain**;**

Servicio Andaluz de Salud, Complejo hospitalario Universitario de Granada (Hospital Universitario San Cecilio), Spain**;**

Servicio Andaluz de Salud, Complejo hospitalario Universitario de Granada (Hospital Virgen de las Nieves), Spain**;**

Servicio Andaluz de Salud, Hospital Regional Universitario de Málaga, Spain**;**

Università degli studi di Milano, Milan, Italy**;**

Hospitaux Universitaires de Genève, Switzerland**;**

University of Szeged, Szeged, Hungary**;**

Charite, Berlin, Germany**;**

Andalusian Public Health System Biobank, Granada, Spain

### Cardiovascular score (CV) estimation

We estimated the cardiovascular (CV) score based on the presence of cardiovascular-related symptoms including: Arrythmia, Coronary artery disease, Hypertension, Pericarditis, Pulmonary arterial hypertension by right-heart catheterization, Pulmonary hypertension on Echo, Valve lesions, Arterial/Venous thrombosis, Gangrene of the fingers, History of Raynaud’s phenomenon, History of recurrent miscarriage or pregnancy complications and Ischemic digital ulcers/Pitting scars. For the current study, we didn’t discriminate between particular cardiovascular symptoms and comorbidities, but estimated the combined score (CV score). According to clinical data, the particular CV feature could be known for the patient in the past or present at the moment of blood sampling. We assigned the following values for each patient: “2”, if CV symptom or comorbidity is present, “1”, if it was described for this patient in the past, “0” for absence or unknown status. For each patient CV score these values were summarized. CV score among studied individuals varied from 0 to 16.

### IgM measurement by ELISA

Levels of IgM antibodies against PC^14,17^ or MDA^15,26^ were determined by enzyme-linked immunosorbent assay (ELISA), as described in detail previously^14,15,17,26^. PC was conjugated with bovine serum albumin (BSA) and MDA was conjugated with human serum albumin (HSA; (Sigma-Aldrich AB, Stockholm, Sweden)). NUNC immune plates were coated with PC-BSA or MDA-HSA at 10 μg/mL, overnight. Plates were washed with PBS containing 0.5% tween (PBST) four times and blocked with 2% BSA for 90 min at room temperature (RT). Plates were washed again. Standards and serum were added at 100 μl/well in duplicates at 1:100 dilution, which was further incubated for two hours at RT. Further, secondary antibodies at (1:3500) for PC-BSA and (1:1500) for MDA-HSA were added and incubated at RT for two hours. The color was developed with pNPP substrate by incubating for 75 min at RT. Finally, the reaction was stopped with 3 M NaOH and the plates were read with spectrophotometer at 405 nm. The Relative Unit (RU) were measured by the formula given below,

$$\frac{=({\rm{A}}{\rm{b}}{\rm{s}}{\rm{o}}{\rm{r}}{\rm{b}}{\rm{a}}{\rm{n}}{\rm{c}}{\rm{e}}\,{\rm{o}}{\rm{f}}\,{\rm{s}}{\rm{a}}{\rm{m}}{\rm{p}}{\rm{l}}{\rm{e}})-({\rm{A}}{\rm{b}}{\rm{s}}{\rm{o}}{\rm{r}}{\rm{b}}{\rm{a}}{\rm{n}}{\rm{c}}{\rm{e}}\,{\rm{o}}{\rm{f}}\,{\rm{b}}{\rm{l}}{\rm{a}}{\rm{n}}{\rm{k}})\ast 100}{({\rm{A}}{\rm{b}}{\rm{s}}{\rm{o}}{\rm{r}}{\rm{b}}{\rm{a}}{\rm{n}}{\rm{c}}{\rm{e}}\,{\rm{o}}{\rm{f}}\,{\rm{S}}{\rm{t}}{\rm{a}}{\rm{n}}{\rm{d}}{\rm{a}}{\rm{r}}{\rm{d}})-({\rm{A}}{\rm{b}}{\rm{s}}{\rm{o}}{\rm{r}}{\rm{b}}{\rm{a}}{\rm{n}}{\rm{c}}{\rm{e}}\,{\rm{o}}{\rm{f}}\,{\rm{b}}{\rm{l}}{\rm{a}}{\rm{n}}{\rm{k}})}$$

### Studies of T-regulatory cells

Generation of Tregs were performed as described previously^28^. Briefly, peripheral blood mononuclear cells (PBMCs) were isolated from healthy donors, according to the manufacturer’s protocol (Ficoll-Paque PLUS, GE Healthcare, Buckinghamshire, United Kingdom). The cells were added to a tissue culture plate pre-coated with anti-CD3 antibody (10 μg/mL) (eBioscience, CA, USA), together with soluble anti-CD28 antibody (1 μg/mL) (eBioscience, CA, USA), IL-2 (10 ng/mL), TGF-β1 (10 ng/mL) (Immuno Tools, Friesoythe, Germany), in culture medium for six days and Tregs were stimulated with phorbol myristate acetate (50 ng/mL) and Ionomycin (1 μg/mL) (Sigma-Aldrich, St Louis, USA) 5 h on the harvest day. Anti-PC and anti-MDA IgM (5 μmol/L) and their respective flow through (FT) were added one day before harvest. PBMCs were resuspended in FACS buffer. For Treg staining, cells were incubated with human Treg cocktail (CD4/CD25/CD127) (BD Bioscience, CA, USA). Experiments were performed with the BD LSRFortessa™ cell analyzer (BD Bioscience, CA, USA) and analysed using flowJo.

### Preparation of IgM anti-PC and anti-MDA

Anti-PC and anti-MDA were extracted as previously described^15,26^. Briefly, PC-BSA and MDA-HAS, 1 mg/mL was coupled to Hitrap NHS column (GE Healthcare, Sweden). Human IgM (Sigma Aldrich, Israel) was passed through Sepharose column coupled with PC-BSA and MDA–HSA. Unbound IgM considered as non-anti-MDA or non-anti-PC (mentioned as flow through, FT) was collected by washing the columns with binding buffer followed by elution with 0.1 mol/L glycine–HCl, elution buffer. The eluted antibodies were desalted in PD-10 columns (GE Healthcare, UK) and concentrated by a Centriprep centrifugal filter (Millipore, Ireland). After filtration through a 0.22-lm filter (Sarstedt, Germany), extracted antibodies were stored at −20 °C.

### IgM protein and peptide characterization

Sample preparation, LC-MS/MS analysis and data processing of the anti-MDA and the anti-PC IgM samples have been described elsewhere^15,28^. Data processing was performed using Spotlight proteomics approach^29^.

Note, that all samples were prepared and analyzed at the same occasion, thus the differences between anti-PC and anti-MDA are more likely due to true biological differences and not to differences according to variation in sample preparation and instrumentation. Briefly, triplicates of the samples: anti-MDA versus flow through [FT] deprived from anti-MDA as well as quadruplicates of the samples: anti-PC versus FT deprived from anti-PC were prepared. Samples were reduced (20 mM dithiothreitol, 30 min, 56 °C), alkylated (66 mM iodoacetamide for 30 min, in the dark) and digested with trypsin (at a ratio of 1:30 enzyme:protein, 37 °C overnight). Peptides were desalted using C18 StageTips (Thermo Scientific), dried in a SpeedVac and resuspended in 0.1% formic acid and 1% acetonitrile. Samples were injected onto a reversed phase 15 cm column (PepMap, C18, 3μm, 100 Å) in 1 µg aliquots using a nano-liquid chromatography system Ultimate 3000 connected to a Fusion Orbitrap mass spectrometer (both - ThermoFisher Scientific). Briefly, survey mass spectra were acquired in the range of *m/z* 300–1700 with a nominal resolution of 120,000. Precursor ion HCD and ETD fragmentation was performed. Raw data processing was performed using the Spotlight proteomics approach which combines quantitative proteomics analysis of de novo sequenced peptides and known peptide sequencing. The abundances of IgM peptides were normalized so that the total abundance was the same (100%) in all samples.

Differences between anti-PC, anti-MDA and non-specific IgM peptides were tested using 2-tailed Student t-test with equal or unequal variance depending upon F-test. Principal component analysis (PCA) and Orthogonal Projections to Latent Structures Discriminate Analysis (OPLS-DA) were performed using SIMCA 14.0 (Umetrics, Umeå, Sweden) following mean centering, log scaling, and univariate scaling.

### Statistical analysis

The antibody levels were used for cases vs controls and cross-disease analysis. Statistical differences were estimated using parametric tests, using 2-tailed t-test with equal or unequal variance depending upon F-test. We applied Shapiro Wilk and Jarque–Bera tests to check the data for normality. The additional non-parametric Epps-Singleton test was carried out for distributions comparison. Percentiles were estimated using total dataset for all cases and healthy controls for each antibody. The correlation between antibody level and cardiovascular score (CV) was estimated using Spearman’s rank test.

For analysis of the level changes of both anti-PC and anti-MDA for MCTD the samples were characterized according to IgM level percentiles and the fraction of samples was estimated for heatmap construction.

Results of experiments with Treg polarization are expressed as mean ± standard error of mean. Effects of IgM anti-PC, anti-MDA or control antibodies were compared by two-tailed paired t-test. For all statistical tests a p < 0.05 was considered significant. Correlation between different runs of antibodies was calculated by Spearman correlation.

### Ethics approval and consent to participate

The study was performed in accordance with the Declaration of Helsinki and was approved by ethical committees at each site for the respective sub-cohorts. All subjects gave informed written consent before entering the study.

## Results

### Characteristics of PRECISESADS patients

The characteristics of PRECISESADS patients are presented in Table 1. Although the diseases have different clinical manifestations, they are highly prevalent in females (70.2%-92,4%). The patients have higher CV scores compared to the controls. IgMs against PC and MDA were measured for all the patients and control, and their Relative units, were presented as seen in Table 1. Since these diseases were female biased, we analyzed female-only subset. Intra-assay variability was <10%. When 288 samples were run two times separately, the correlation, R, was 0.988 for IgM anti PC and 0.978 for IgM anti-MDA.

**Table 1:** Dataset of determinations of IgM anti-PC and anti-MDA.

	Samples	Males	Females	Male,%	Cardiovascular comorbidities score, average (min – max)	Average age, all	Average age, males	Average age, females	Anti-PC IgM units (mean ± SD)	Anti-MDA IgM units (mean ± SD)
MCTD	77	11	66	14.3	3.62 (0–15)	50.4 (18–80)	56.3 (45–74)	49.4 (18–80)	61.51 ± 38.72	62.88 ± 33.31
PAPs	65	20	45	30.8	2.98 (0–13)	48.0 (25–79)	52.7 (26–67)	49.9 (25–79)	87.85 ± 44.26	97.59 ± 52.16
RA	354	77	277	21.8	1.32 (0–10)	58.0 (24–87)	60.3 (28–83)	57.4 (24–87)	88.53 ± 43.68	84.34 ± 45.21
SjS	324	21	303	6.5	1.72 (0–10)	58.3 (22–93)	59.4 (26–93)	58.2 (22–86)	68.73 ± 38.91	71.38 ± 37.95
SLE	374	30	344	8.0	2.33 (0–10)	45.8 (19–87)	50.0 (23–77)	45.5 (19–87)	68.27 ± 36.54	76.36 ± 44.35
SSc	331	53	278	16.0	4.84 (0–16)	58.2 (24–86)	55.4 (34–84)	58.7 (24–86)	76.31 ± 35.90	77.04 ± 39.96
UCTD	118	9	109	7.6	2.10 (0–10)	52.7 (19–90)	59.7 (44–80)	52.1 (19–90)	76.11 ± 43.86	83.01 ± 51.82
Control	515	111	404	21.6	0.23 (0–4)	47.6 (20–85)	46.3 (25–78)	48.0 (20–85)	73.86 ± 34.25	69.34 ± 34.24

### Anti-PC and anti-MDA levels among cases and controls

**Figure 1:**
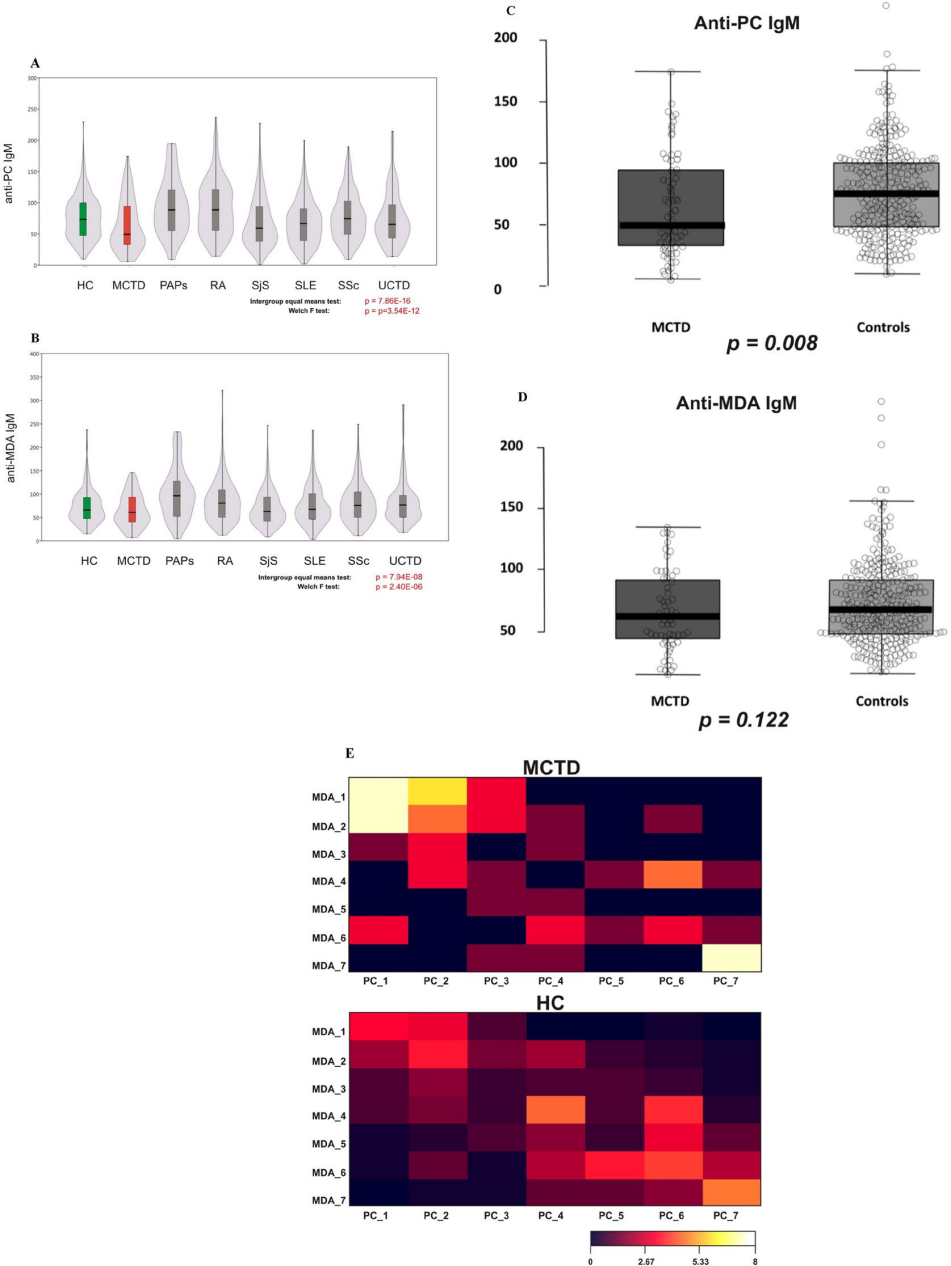
(**A**) IgM Anti-PC in cross sectional cohort, PRECISESADS. There were clear differences between different diagnoses. Low levels were observed in SLE, MCTD and SjS. The box plot for anti-PC IgM levels for all systemic rheumatic diseases and controls. Box limits indicate the 25th and 75th percentiles, centre lines indicate median values, whiskers extend 1.5 times the interquartile range from lower and upper percentiles. (**B**) A different profile was observed in IgM Anti-MDA. Not a clear difference between different diagnoses as for IgM anti-PC. A pairwise t-test was done for any two given diseases, orange boxes represent FDR-corrected p-values <0.005, light orange boxes represents p(_FDR_) < 0.05. Violin and box plots at A, B are shown for female-only data subset. The healthy control group is highlighted in green, the MCTD in red. Inset (**C,D**): anti-PC in MCTD – striking differences as compared to controls. For MCTD case the comparison to healthy controls is presented. Box plot shows significant decrease of anti-PC IgM in MCTD (**C**). No striking differences were detected for anti-MDA IgM antibodies (**D**). T-test p-values shown below boxplots. (**E**) The heatmap based on the fraction of samples for each percentile according to anti-PC and anti-MDA IgM level for MCTD and healthy controls. Numbers correspond to the following percentiles: 1 (<=10%), 2 (10–25%), 3 (25–33%), 4 (50–66%), 5 (66–75%), 6 (75–90%), 7 (>90%). Color scheme for the corresponding fraction (%) of samples is shown below.

Different systemic rheumatic diseases demonstrated variant distribution of anti-PC and anti-MDA levels (Fig. 1A–E). IgM levels were distributed normally in the total dataset as well as in the healthy controls and all diseases combined subsets (Shapiro Wilk p(normal) for anti-PC/anti-MDA = 2.16E-20/5.63E-29, 1.34E-25/8.57E-30 and 1.33E-12/4.54E-07, respectively, Jarque-Bera p(Monte-Carlo) = 0.0003 for disease-only anti-PC and 0.0001 for other datasets). The most prominent changes in mean values in comparison with both healthy controls and other diseases were detected for MCTD with more pronounced level decrease for anti-PC (p = 0.008) than for anti-MDA (p = 0.122). Additional non-parametric Kruskal-Wallis test for the MCTD subset (65 samples) also supported importance of anti-PC IgM for this disease (Dunn posthoc corrected p (same) = 0.005559, in contrast to 0.1777 for anti-MDA). In the cross-sectional data, mostly the anti-PC mean level was significantly different between disorders or HC-disorders in pairwise comparison. Low levels of IgMs were the most prevalent in MCTD, both anti-PC and anti-MDA. Also IgM low levels were more frequent in SjS, SLE, SSc and UCTD, more for anti-PC than for anti-MDA (Suppl. Fig. 1). Variance based tests shown more significant differences in anti-PC IgM than for anti-MDA for most of diseases, including MCTD, SjS, RA and SLE (Fig. 1). Low levels of both anti-PC and anti-MDA in the same samples were prevalent in MCTD and SLE but not in HC and were also detected in other disease cohorts, SLE in particular (Fig. 1E).

### Cardiovascular co-morbidities

**Figure 2:**
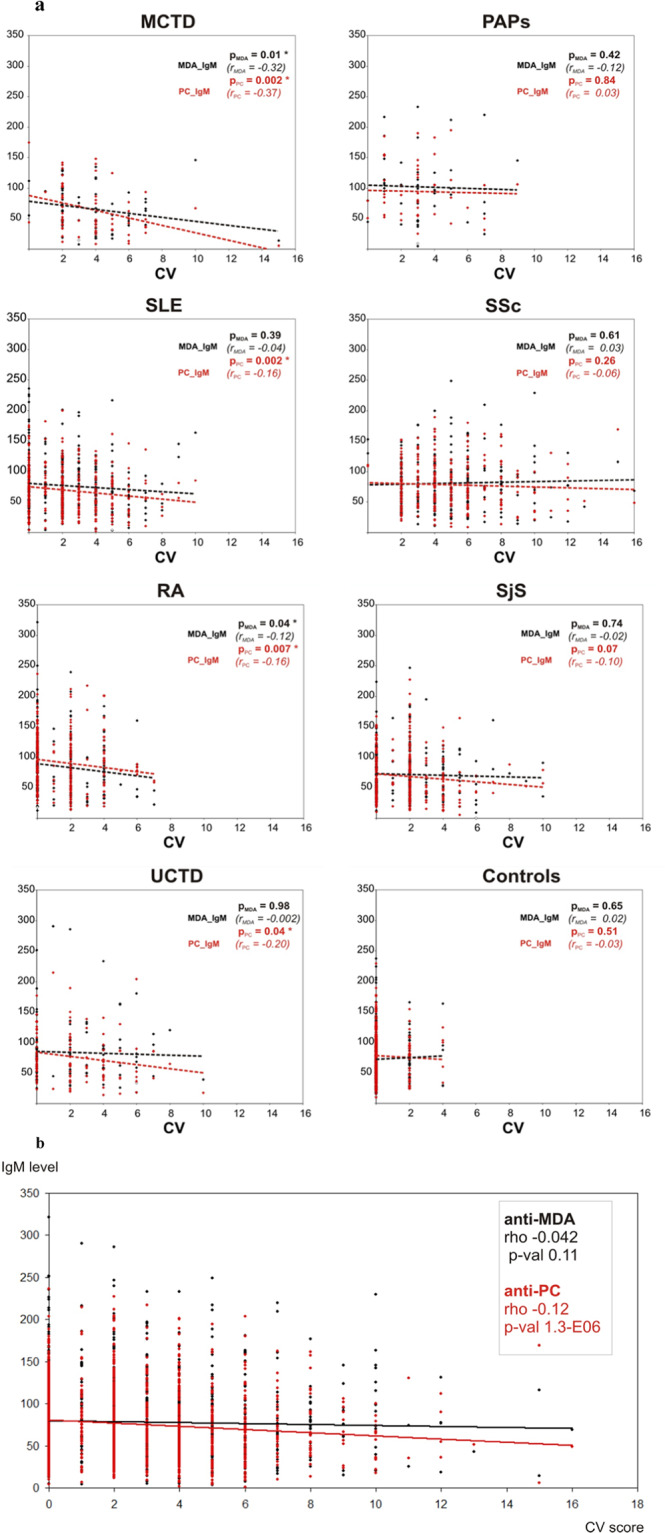
Correlation analysis between cardiovascular score and anti-PC or anti-MDA antibody levels. **(a)** The levels of anti-PC and anti-MDA IgM compared for each disease independently with the cardiovascular score. Individual values are shown as dots with the linear trend lines indicated. Spearman’s rank correlations were estimated between these values. Negative correlation between IgM level and CV was detected for anti-PC but not for anti-MDA for MCTD, SLE, UCTD and RA. No significant correlations were shown for SSc, PAPs and SjS subsets. The total all-disease cohort also demonstrated significant negative correlation between anti-PC IgM and CV (rho −0.128, 2-sided p-value 1.27e-06), but not for anti-MDA (rho −0.042, 2-sided p-value 0.106). **(b)** Cardiovascular comorbidities and symptoms for all cases. The levels of anti-PC and anti-MDA IgM compared for all cases of systemic autoimmune diseases with the cardiovascular score. Individual values are shown as dots with the linear trend lines indicated. Spearman’s rank correlations were estimated between these values. Negative correlation between IgM level and CV was detected for anti-PC but not for anti-MDA.

The frequent cardiovascular comorbidities were described for patients as shown in Fig. 2. We estimated the cardiovascular (CV) score based on the presence of cardiovascular-related symptoms and that it correlates with IgM anti-PC and anti-MDA. We demonstrate that for the combined patient cohort anti-PC IgM is significantly negatively correlated with the CV score, but not anti-MDA (Spearman’s rank correlation p-value 8.3E-05, 8.6-E07 and 0.64). Anti-MDA level correlated with CV score only for MCTD and RA, but not with other diseases. In contrast, for anti-PC IgM this correlation was significant for MCTD, RA, SLE and UCTD but not the healthy controls. Presented data are for females only. Males were few in diseases studied, but when included, data were similar as for women (data shown).

### Effects of IgM anti-PC and anti-MDA IgM on the induced-Treg proportion in PBMCs of Healthy donors

**Figure 3:**
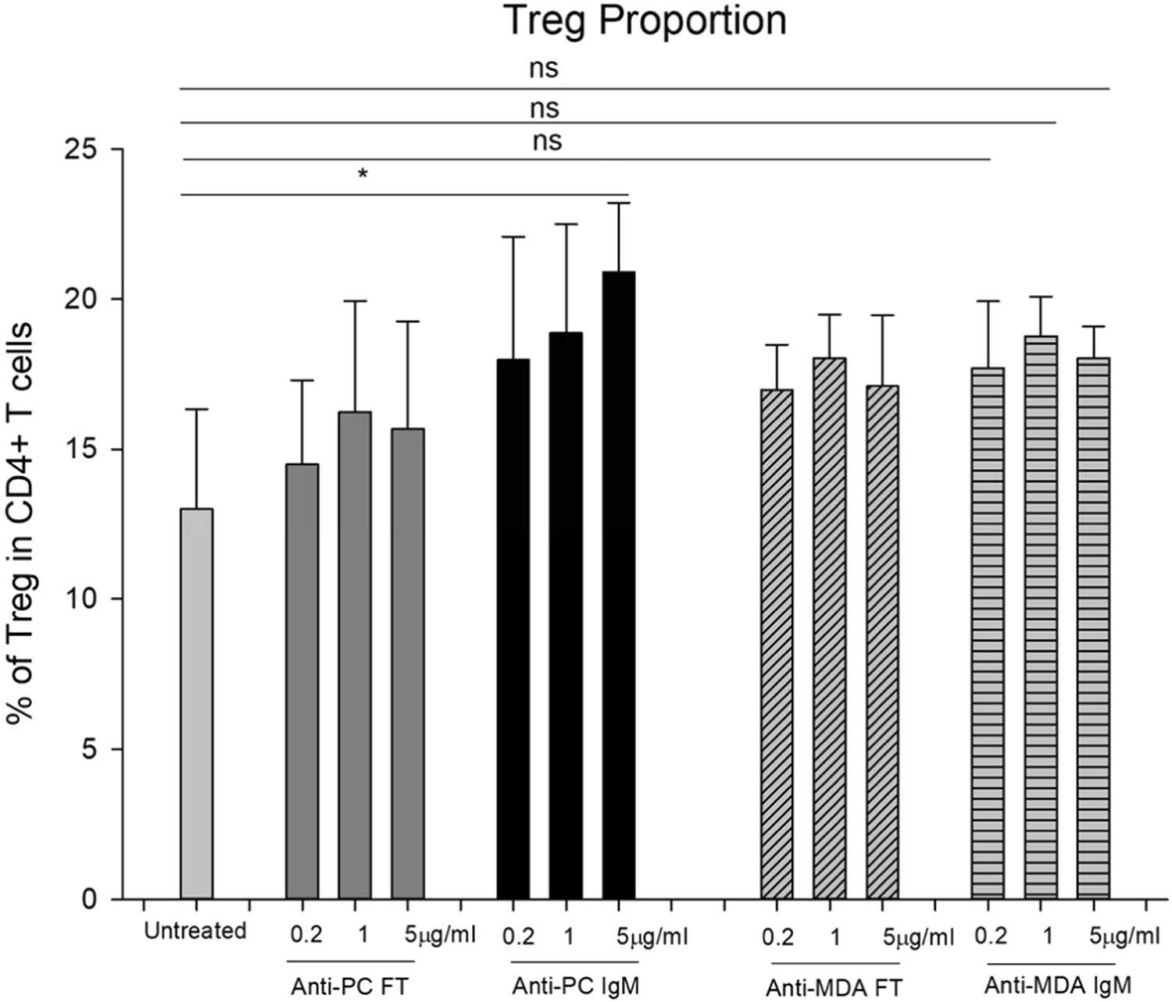
The proportion of Treg (CD4^+^CD25^+^CD127 ^dim/-^) cells in CD4^+^T cells from the Buffy Coat of healthy donors. CD25 and CD127 expression were determined by flow cytometry analysis in CD4^+^T cells after 6 days culture with Treg-polarizing cytokines, with PMA and Ionomycin stimulation. Anti-PC flow, Anti-PC IgM, Anti-MDA flow and Anti-MDA IgM were added one day before harvest. The percentage ±SD of three different cultures performed. Differences between Treg cells Anti-MDA IgM treatment showed statistically significant. (p < 0.05). Differences between Treg cells Anti-MDA IgM treatment showed no statistically significant effects. Effects of anti-PC and anti-MDA on Treg polarization.

To investigate if IgM anti-MDA promotes polarization of Tregs in a similar way as we have previously reported for anti-PC, we cultured PBMC from three healthy blood donors, and treated them in parallel either with anti-PC, anti-MDA and Ft (for the respective antibody). While IgM anti-PC increased Tregs upon anti-PC IgM addition as reported previously (the three experiments were included in the pooled results presented before), but anti-MDA did not promote any significant differences in Treg proportions (Fig. 3).

### Proteomic comparative analysis of polyclonal anti-PC and anti-MDA variable regions

We have previously published quantitative LC-MS/MS peptide sequencing data from the anti-PC variable region^28^ and the anti-MDA variable region^15^.

Herein we reanalyzed the data to compare and identify potential similarities and differences between the two antigens specific IgM peptide profiles with focus on peptides derived from the variable region (i.e. peptides identified as perfect matches to known variable region peptide sequences or derived from de novo sequences with sequence homology to the variable regions).

In line with findings from our previous studies using quantitative LC-MS/MS peptide sequencing, we could demonstrate that anti-MDA and anti-PC contained significantly lower amounts of lambda chain sequences compared to the corresponding flow through fractions (deprived from the antigen specific IgM). When comparing the identified variable peptide sequences (n = 963) from anti-MDA, anti-PC or their corresponding FTs, approximately 50% (n = 471) were not detectable in the anti-MDA and anti-PC IgM.

**Figure 4:**
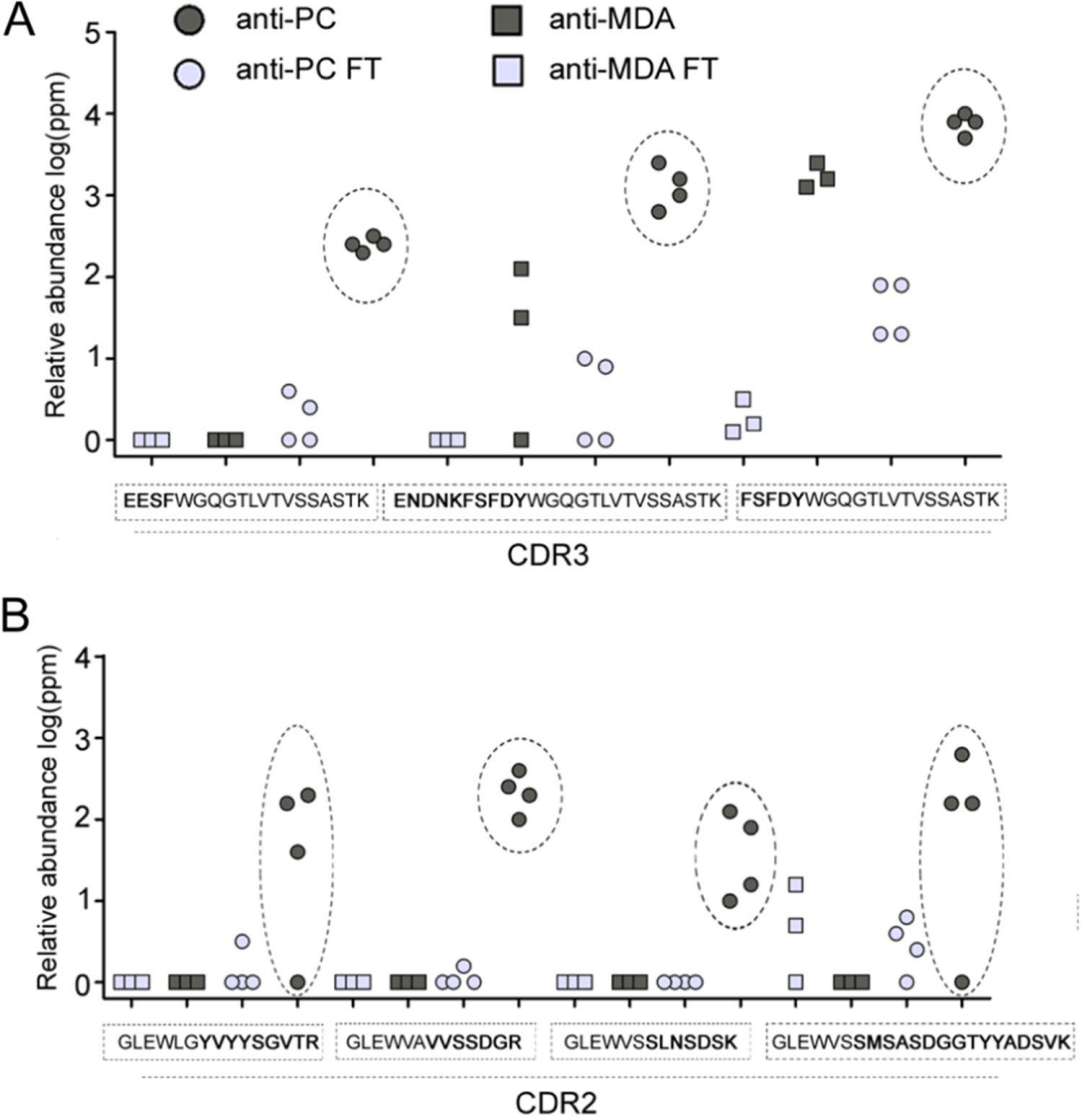
Proteomics analysis. Similarities and differences between the polyclonal anti-PC and anti-MDA IgM variable regions. Differences in the heavy variable chain CDR2 and CDR3 regions between polyclonal anti-PC IgM and non-anti-PC IgM (flowthrough, FT) as well as anti-MDA and anti-MDA-FT samples. The data was generated via quantitative proteomics *de novo* sequencing analysis. Numbers are reported as log(ppm) of the relative variable region peptide distributions in respective sample. (**A**) Peptides from the HV CDR3 region that were elevated in the anti-MDA IgM and/or anti-PC samples. (**B**) Peptides from the HV CDR2 region that were elevated in the anti-PC IgM samples.

**Figure 5:**
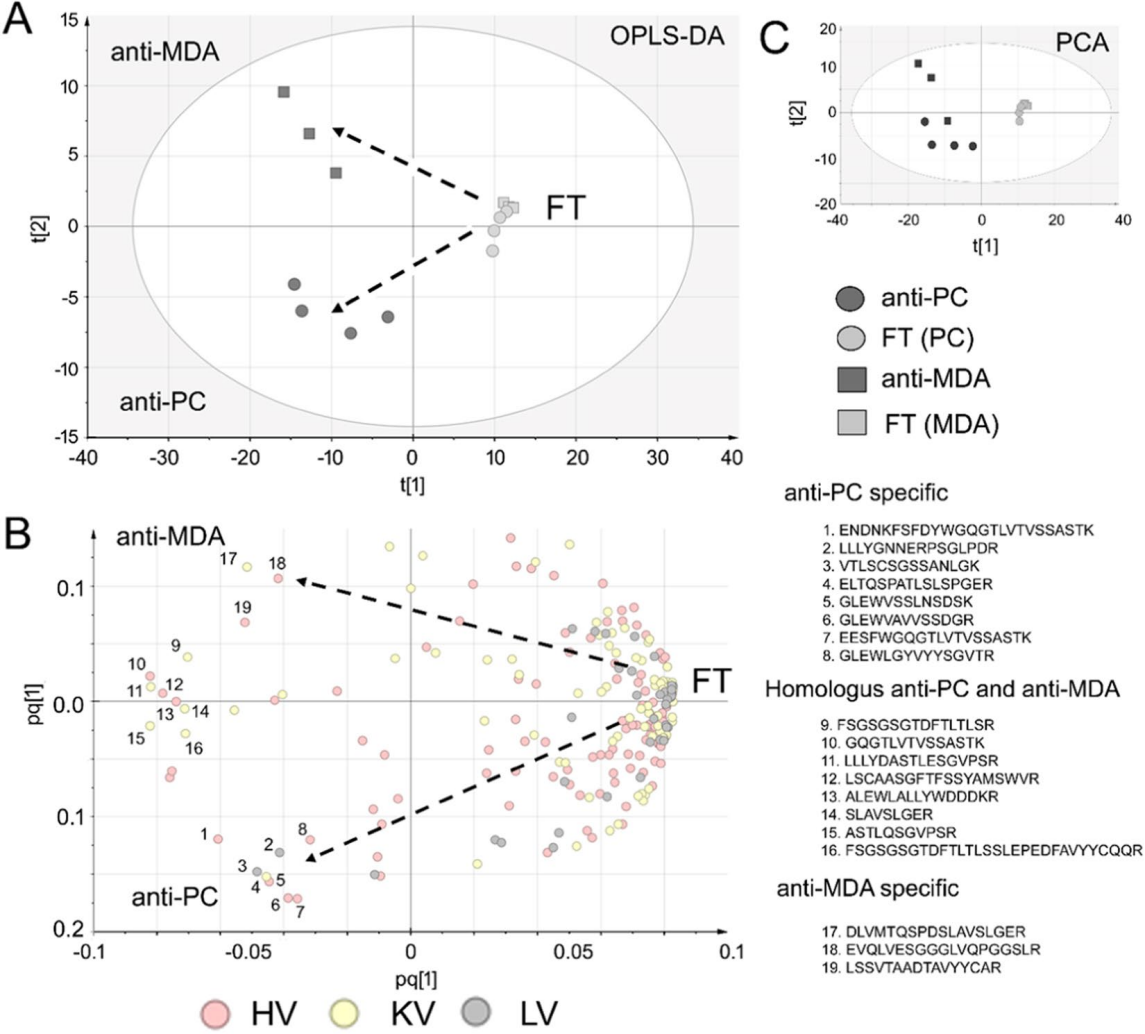
Differences in the heavy variable chain regions between the anti-PC, anti-MDA and FT samples as determined via multivariate data analysis using the proteomics *de novo* sequencing quantitative data. (**A**) Scores plot of the OPLS-DA multivariate analysis of the anti-PC samples (group 1), anti-MDA samples (group 2) and anti-PC-FT and anti-MDA-FT samples (group 3) using heavy variable, lambda variable and kappa variable chain peptides that were identified in both FT and anti-PC or anti-MDA samples or identified in anti-PC or anti-MDA only. The generated model (R2 = 0.9, Q2 = 0.7, p = 0.0003) contained two predictive components (axis x and axis y). The scores plot shows distinct separation between the FT and antigen specific IgM samples along the x-axis and separation between the anti-MDA and anti-PC IgM samples along the y-axis. (**B**) Loading plot showing how the peptides correlate with respective subgroup. From the plot it is evident that the majority of peptides correlate with the FT but that particular peptides are specific for anti-PC (nr. 1–8) and anti-MDA (nr 17–19). Furthermore, a number of peptides (those negatively correlating with the FT and oriented between anti-MDA and anti-PC), are peptides that were elevated in both anti-PC and anti-MDA IgM but low in abundance (or missing) in the FT (nr. 9–16). Note that we have previously described nr 11–17 and 19 as anti-MDA specific^15^. (**C**) The corresponding PCA scores plot of the data.

Among anti-MDA and/or anti-PC IgM peptides, specific CDR peptide sequences (ENDNKFSFDYWGQGTLVTVSSASTK and FSFDYWGQGTLVTVSSASTK) in both anti-PC and in anti-MDA were raised significantly as compared to Ft, Fig. 4A. Furthermore, particularly the anti-PC specific IgM contained HV, KV and LV peptide sequences that appears to be specific for this type as demonstrated both by univariate (as shown in Fig. 4B and in multivariate (as shown in Fig. 5) data analysis. Particularly the multivariate data which was constructed by PCA, (Fig. 5C) and OPLS-DA (Fig. 5A) differentiated the antigen specific IgM to the FT samples along component 1 (x-axis) and the anti-PC and anti-MDA samples along component 2 (y-axis). From the OPLS-DA loading plot the variable region peptide sequences that were abundant in the anti-MDA, anti-PC or both anti-MDA/anti-PC were then identified. The most prominent differences between anti-PC and anti-PC-FT as well as anti-MDA and anti-MDA-FT, respectively are shown in Supplemental Table 2.

## Discussion

We here report that anti-PC IgM is significantly lower among patients with MCTD as compared to controls, and also that different systemic autoimmune diseases show different anti-PC and, in less extent, anti-MDA IgM level variation. Anti-PC concentration was remarkably different for MCTD, RA, SLE and SjS in comparison with healthy controls. Clear differences were found between anti-PC IgM in patients with RA, PAPs and MCTD, with higher levels among the former two diagnoses as compared to MCTD. Several diseases were characterized with increased fraction of samples with extremely low anti-PC IgM level: SjS, SLE, UCTD. This effect was the most pronounced for MCTD, both for anti-PC and anti-MDA.

MCTD is a relatively rare autoimmune disease and it’s sheer existence as a distinct disorder and diagnostic criteria are still debated^30,31^. Our findings could favour the notion that it indeed is a disease of its own. Our findings are also in line with a recent report where increased risk of cardiovascular disease was present in MCTD^32^.

The prevalence of low anti-PC IgM in SLE is in line with our previous findings which have been largely confirmed by others^12–14,16–25^.

Anti-PC analysis for MCTD, SSc, UCTD and SjS is presented here for the first time and detected association are thus new. Relatively little is known for these antibodies putative role in RA. We didn’t detect lower anti-PC level samples in RA, moreover, this level was higher than in controls. Anti-PC IgM can still have some protective properties in this pathology but current cross-section design is a limitation. Previously we detected low anti-PC levels for non-responders to treatment to biologics in RA^18^. Other published evidence of a role as protection marker of IgM anti-PC comes from studies on vasculitis and even osteoarthritis^24^.

We also found that anti-PC, but not anti-MDA IgM is negatively associated with broadly defined cardiovascular symptoms and comorbidities among whole group of patients and, specifically, in MCTD, RA, SLE and UCTD. This finding is in line with our previous results on SLE-associated cardiovascular complications^16,17^.

The important question is if the associations with IgM anti-PC as a protection marker also represent underlying mechanisms which could cause or have secondary effects on the disease conditions. The present cross-sectional study design does not allow conclusions about cause, and in fact, low levels of anti-PC could have different causes. Low anti-PC levels could be caused by the disease and not the other way around, even though our previous findings, where low levels predicted disease development, to some extent argues against this possibility. For example, increased oxidation of LDL is a feature of SLE, where OxLDL as determined by PC-exposure was increased among patients as compared to controls and also among SLE-patients with CVD as compared to those without^33^. Thus, both atherosclerosis and SLE, could in principle trigger consumption of anti-PC. It is also possible that immune complexes could play a role, which contain the antigens studied^34^. Still, even if consumption and immune complex formation contributes, low levels of antibodies still predict disease. We have demonstrated that levels of IgM anti-PC are positively associated with anti-PC production by B-cells. We suggest that this argues against consumption being a cause of low levels, though it’s contribution cannot be completely ruled out^35^.

There are several lines of evidence in support of IgM anti-PC being not just a disease marker but also a potential contributing factor. The risk of CVD in SLE is increased, which is likely to be related to such risk factors as being oxidation leading to PC-exposure and being low anti-PC levels^33^. Anti-PC could be protective by inhibiting OxLDL uptake into inert macrophage-derived foam cells^36^, and also could inhibit cell death caused by a major OxLDL component^37^. Further, anti-PC is anti-inflammatory by inhibiting the effects of inflammatory phospholipids^17^. We previously reported that IgM anti-PC increase clearance of dead and dying cell^26^, this notion is in line with animal experiments showing increased uptake of apoptotic cells by IgM anti-PC^38^. Inefficient dead cells clearance may contribute to SLE and likely to other systemic autoimmune disorders, at least such as MCTD^39^. Recently we demonstrated yet another mechanism which potentially could be protective both in atherosclerosis and CVD, as well as in rheumatic diseases. Hence, we found that IgM anti-PC promotes polarization of T regulatory cells, both in from atherosclerotic plaques and SLE-patients^28^. In SLE, Tregs were lower than among controls which is in line with previous studies where Tregs were negatively associated with SLE^40^. In an interesting recent study, IgM anti-PC was confirmed to be depressed in SLE and associated with atherosclerosis in this condition. Further, Low IgM anti-PC was associated with high triglyceride and low HDL – a pro-inflammatory lipid profile - and also to low Treg levels. This finding is highly compatible with the notion that IgM anti-PC promote Treg polarization^25^.

In our study the levels of Tregs were normalized by IgM anti-PC treatment which was raised to the same levels as healthy controls^28^. Tregs are known to be decreased in MCTD and could be a contributing factor in disease pathogenesis and development^41^. In principle, low anti-PC levels could thus contribute to low Treg-levels. Low or dysfunctional Tregs have also been implicated in SjS^42^ and in SSc. However, even though several publications indicate low levels in SSc, this is more controversial and may be related more to dysfunctional Tregs than to their reduced levels^43^.

In contrast to IgM anti-PC, IgM anti-MDA did not differ much between groups^26^. We still see herein low anti-MDA levels in patients, however, strongest findings are for anti-PC. Previously we reported anti-MDA negative associations with atherosclerosis measurements, which was not performed in the present study. Still, among RA and MCTD patients, prevalence of CV-comorbidity was negatively associated with IgM anti-MDA. Of note, in the present study, we had broad definitions of CV as compared to the previous study which focused on SLE, atherosclerosis and CVD^26^.

It is possible that IgM anti-PC, and potentially also IgM anti-MDA has a role among some SLE-patients, in subgroups, e.g. those with CVD-risk or established CVD and with atherosclerosis. Further studies are needed to clarify the role of anti-PC and anti-MDA as risk/protection markers in SLE and also in the other autoimmune diseases studied, especially MCTD and SS where we here report significant associations. Anti-MDA has some properties in common with IgM anti-PC, such as decreased uptake of OxLDL in macrophages^44^ which could be of importance for atherosclerosis. Thus, this could lead to growth of atherosclerotic plaques with accumulation of macrophage-derived foam cells filled with OxLDL, becoming inert and undergoing cell death, through apoptosis or necrosis. Our preliminary data indicate that anti-MDA has anti-inflammatory properties, inhibiting effects of MDA-epitopes (unpublished). Anti-MDA is also known to increase uptake of human apoptotic cells^26^.

Interestingly, in contrast to IgM anti-PC, IgM anti-MDA did not have the ability to promote polarization of Tregs. There is thus a possibility that this difference between the antibodies could explain the apparently higher protective potential of IgM anti-PC in our study.

By use of proteomics analysis, we also demonstrate interesting differences between IgM anti-PC, anti-MDA and control IgM. We have previously published quantitative LC-MS/MS peptide sequencing data for anti-PC^28^ and anti-MDA variable regions^15^. Herein we reanalyzed the data to compare and identify potential similarities and differences between the two antigens specific IgM peptide profiles with focus on peptides derived from the variable region. Both anti-MDA and anti-PC contained significantly lower amounts of lambda chain sequences compared to the corresponding flow through fractions (deprived from the antigen specific IgM). We also demonstrate that the antigen specific IgM are more homogeneous than the control IgM (FTs) and contain elevated levels of specific IgM variants. Noteworthy, among the anti-MDA and/or anti-PC IgM peptides, we could identify significantly elevated levels (compared to the FT) of specific CDR peptide sequences. These findings are thus in line with the somewhat different clinical and functional properties of IgM anti-PC and anti-MDA.

It has been described earlier that MDA-modified LDL is scavenged by monocytes/macrophages in atherosclerotic plaques and thus could play a role in atherogenesis^45^. One dominant epitope in MDA-protein adducts is acetaldehyde that forms stable dihydropyridine (4-methyl-1,4-dihydropyridine-3,5-dicarbaldehyde), which in turn modifies ubiquitous and essential amino acid lysine to a stable product which is implicated in atherosclerosis and other inflammatory conditions^46^.

PC is exposed on oxidized lipids as in OxLDL which is a major component of atherosclerotic plaques. Together with the formation/accumulation of dead cells this results in an ongoing chronic inflammation and thus activated immune competent cells^12,47^. OxLDL is also increased in SLE, and associated with CVD, as determined by PC exposure, and also other plasma components exhibit increased PC in this condition^33^. PC is usually described as a danger associated molecular pattern, DAMP, which is the case also with MDA. These compounds, when exposed, are recognized as danger and something that should be removed. Interestingly, in contrast to MDA, PC is also a pathogen associated molecular pattern, PAMP, being of importance on infectious agents including parasites and nematodes among others. This could represent yet another interesting difference between these types of antibodies. We have proposed a development of the Old friends/Hygiene hypothesis, where lack of exposure to PC-exposing microorganisms and pathogens as nematodes and parasites could lead to lower levels of anti-PC and as consequence lower protection against chronic inflammatory conditions. In this process both lipid oxidation and PC exposure will play a role. We demonstrated that in Kitava, Papua New Guinea, anti-PC is very high, and these diseases are non-existing^48,49^. We also demonstrated that one pathogen implicated could be Treponema, causing Yaws in these areas^50^.

There are several limitations in this study. One is the cross-sectional design, where we cannot draw conclusions about a potential role in predicting disease manifestations in relation to the antibodies studied. Also other IgM antibodies including total IgM could be of interest to study, but in this study we focused on those which appear most promising in relation to disease and outcome. It could also be of interest in further studies to analyse if different carriers for these epitope could make any difference. The experimental studies of antibodies in relation to Tregs are not the main topic of this studies, and larger studies of T cell populations and T reg polarization are needed to confirm this relatively small sample, even though our results were significant.

## Conclusions

Our data indicates that anti-PC but probably not anti-MDA IgM may play a role as a marker in systemic autoimmune diseases such as MCTD, SjS and SLE, and where the negative associations were strongest for MCTD. Our findings also confirm and extend previous observations of negative associations between these IgMs and CVD in autoimmune patients. Our results are compatible also with anti-PC contributing to the diseases per se, since several mechanisms which potentially could be protective have been identified, incuding promotion of Treg polarization. It remains to be seen, however, if raising anti-PC with active or passive immunization is beneficial.

## Supplementary information

Supplementary information

## Data Availability

Data and materials are available upon request.
