# Supplementary material for: IgM antibodies against malondialdehyde and phosphorylcholine in different systemic rheumatic diseases
Source: Sci Rep. 2020 Jul 3;10:11010. doi: 10.1038/s41598-020-66981-z (PMC7335044; doi:10.1038/s41598-020-66981-z)

Supplementary material

IgM antibodies against malondialdehyde and phosphorylcholine in different systemic rheumatic diseases

Divya Thiagarajan[1], Nina Oparina[1], Susanna Lundstrцm[2], Roman Zubarev[2], Jitong Sun[1], the PRECISESADS Clinical Consortium[3], Marta Alarcon-Riquelme[1,3] and Johan Frostegard[1*]

[1]Unit of Immunology and Chronic Disease, Institute of Environmental Medicine, Karolinska Institutet, Stockholm, Sweden

[2]Division of Physiological Chemistry I, Department of Medical Biochemistry and Biophysics, Karolinska Institutet, Stockholm, Sweden

[3]GENYO, Center for Genomics and Oncological Research: Pfizer/University of Granada/Andalusian Government, Parque tecnolygico de la salud, 18016, Granada, Spain

Supplementary table 1. Cross-disease comparison of anti-PC and anti-MDA IgM levels

A. FDR-corrected p-values of pairwise comparisons: Mann-Whitney

MDA_IgM	Control	MCTD	PAPs	RA	SjS	SLE	SSc	UCTD
Control		0.174	0.004	0.004	0.306	0.639	0.042	0.174
MCTD	0.174		0.004	0.008	0.455	0.163	0.027	0.053
PAPs	0.004	0.004		0.095	0.002	0.008	0.042	0.055
RA	0.004	0.008	0.095		0.002	0.027	0.412	0.455
SjS	0.306	0.455	0.002	0.002		0.174	0.008	0.058
SLE	0.639	0.163	0.008	0.027	0.174		0.164	0.306
SSc	0.042	0.027	0.042	0.412	0.008	0.164		0.874
UCTD	0.174	0.053	0.055	0.455	0.058	0.306	0.874	

PC_IgM	Control	MCTD	PAPs	RA	SjS	SLE	SSc	UCTD
Control		0.006	0.024	~.0001	0.001	0.002	0.806	0.327
MCTD	0.006		0.001	<.0001	0.350	0.266	0.006	0.087
PAPs	0.024	0.001		0.806	0.001	0.001	0.047	0.021
RA	~.0001	<.0001	0.806		<.0001	<.0001	0.001	0.001
SjS	0.001	0.350	0.001	<.0001		0.586	0.001	0.177
SLE	0.002	0.266	0.001	<.0001	0.586		0.003	0.342
SSc	0.806	0.006	0.047	0.001	0.001	0.003		0.299
UCTD	0.327	0.087	0.021	0.001	0.177	0.342	0.299	

B. FDR-corrected p-values of pairwise comparisons: two-tailed t-test

MDA_IgM	Control	MCTD	PAPs	RA	SjS	SLE	SSc	UCTD
Control		0.169	0.006	0.002	0.508	0.290	0.020	0.025
MCTD	0.169		0.002	0.002	0.317	0.055	0.011	0.017
PAPs	0.006	0.002		0.091	0.004	0.014	0.035	0.097
RA	0.002	0.002	0.091		<.0001	0.035	0.317	0.850
SjS	0.508	0.317	0.004	<.0001		0.135	0.011	0.035
SLE	0.290	0.055	0.014	0.035	0.135		0.239	0.228
SSc	0.020	0.011	0.035	0.317	0.011	0.239		0.618
UCTD	0.025	0.017	0.097	0.850	0.035	0.228	0.618	

PC_IgM	Control	MCTD	PAPs	RA	SjS	SLE	SSc	UCTD
Control		0.004	0.024	<.0001	0.006	0.003	0.001	0.027
MCTD	0.004		0.002	<.0001	0.474	0.474	0.022	0.117
PAPs	0.024	0.002		0.474	0.002	0.002	0.037	0.002
RA	<.0001	<.0001	0.474		<.0001	<.0001	<.0001	0.002
SjS	0.006	0.474	0.002	<.0001		0.997	0.005	0.172
SLE	0.003	0.474	0.002	<.0001	0.997		0.003	0.165
SSc	0.001	0.022	0.037	<.0001	0.005	0.003		0.552
UCTD	0.027	0.117	0.002	0.002	0.172	0.165	0.552	

Color scheme: ***p<0.05*** ***p<0.005***

Supplementary Table 2. The most prominent differences between anti-PC and anti-PC-FT as well as anti-MDA and anti-MDA-FT, respectively. Numbers are reported as log(ppm) of the relative peptide distribution in respective sample. CDR peptide sequence regions are bolded. Peptides with a ppm <1, were set as 0. – peptide was not detected. p<0.05 (significant) are marked out.

Type	Region	Sequence	anti-PC	anti-PC-FT	anti-MDA	anti-MDA-FT	p-values -anti PC vs		
							anti-PC-FT	anti-MDA	anti-MDA-FT
HV	CDR3	**EESF**WGQGTLVTVSSASTK	2.4±0.1	0.3±0.3	-	-	1.4E-05	-	-
		**ENDNKFSFDY**WGQGTLVTVSSASTK	3.1±0.2	0.5±0.5	1.2±1.1	-	1.1E-04	8.8E-02	-
		**FSFDY**WGQGTLVTVSSASTK	3.9±0.1	1.6±0.4	3.2±0.2	0.2±0.2	1.7E-05	2.1E-03	7.9E-07
	CDR2	GLEWLG**YVYYSGVTR**	1.5±1.1	0.1±0.3	-	-	7.6E-02	-	-
		GLEWVA**VVSSDGR**	2.3±0.3	0.1±0.1	-	-	3.5E-06	-	-
		GLEWVS**SLNSDSK**	1.6±0.5	-	-	-	-	-	-
		GLEWVS**SMSASDGGTY**YADSVK	1.8±1.3	0.4±0.3	-	0.6±0.6	7.7E-02	-	1.9E-01
	FR3	NTLYLQMNSLTTEDTALYYCAK	3.3±0.2	1.1±0.2	2.6±0.1	0.0±0.0	1.2E-05	4.7E-03	9.3E-05
KV	CDR2	**ASTLQ**SGVPSR	4.5±0.2	2.0±0.2	4.5±0.2	1.7±0.2	5.3E-06	8.0E-01	1.2E-05
		LLLY**AASTLQS**GVPSR	2.7±0.2	2.4±0.1	2.7±0.4	2.2±0.3	4.6E-02	9.0E-01	4.0E-02
	FR1	ELTQSPATLSLSPGER	1.1±0.8	-	-	-	-	-	-
LV	CDR1	VTL**SCSGSSANLGK**	2.2±0.3	-	-	-	-	-	-
		**NYVS**WYQQLPGTAPK	2.0±0.6	1.3±0.1	-	1.3±0.1	8.9E-02	-	9.2E-02
	CDR2	LLLY**GNNERPS**GLPDR	2.0±0.8	0.1±0.3	-	0.3±0.5	4.5E-03	-	7.6E-03

Supplement Figure 1. Low levels of anti-PC and anti-MDA IgMs across systemic rheumatic diseases and in healthy controls

For each subset % of samples with the IgM level lower than overall 25th or 10th percentile is shown.

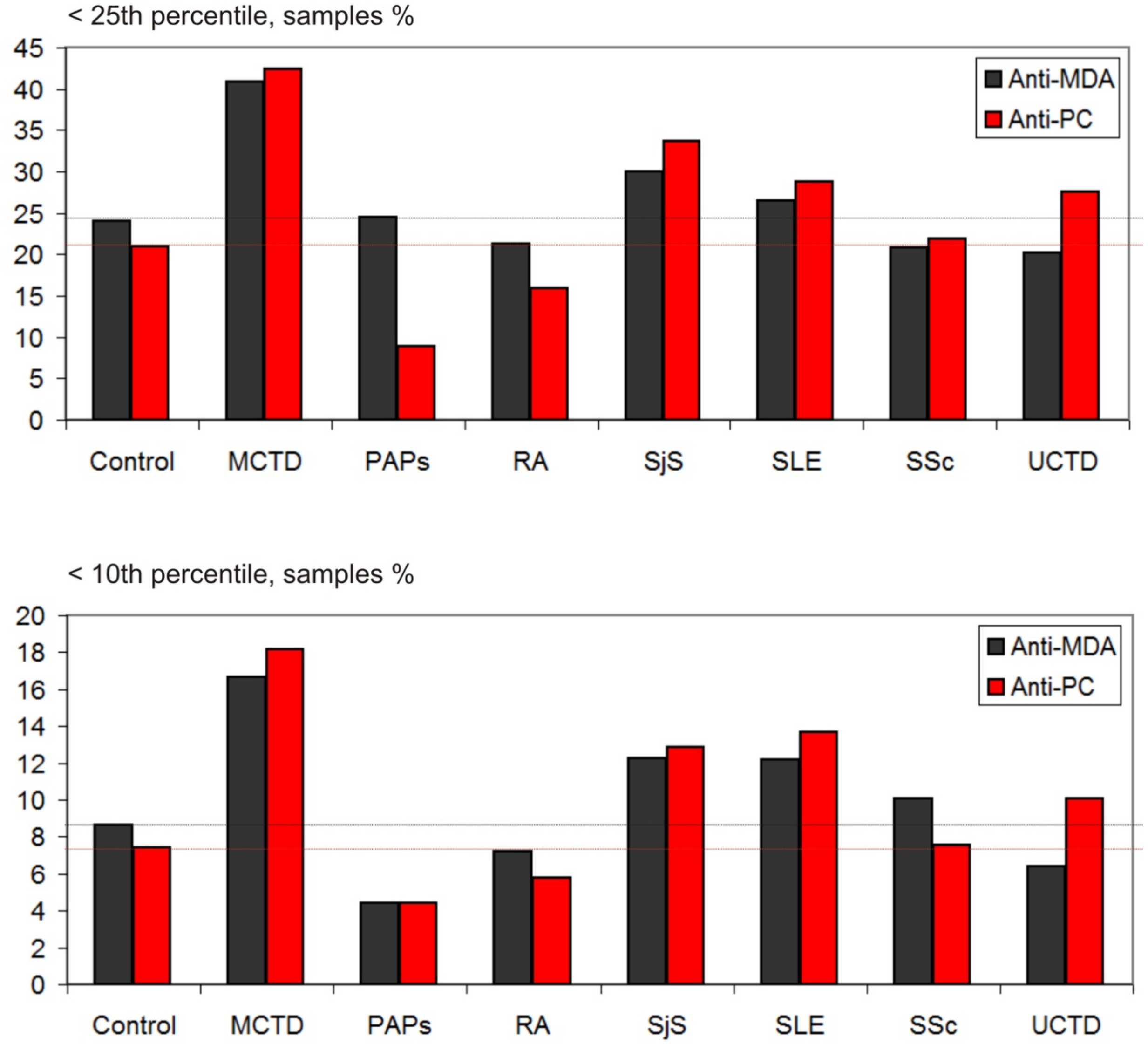

Supplement: Supplementary file 1 — Supplementary information [file 41598_2020_66981_MOESM1_ESM.pdf]
